# Parents’ Beliefs About Play and the Purpose of Preschool Education, Preschoolers’ Home Activity and Executive Functions

**DOI:** 10.3389/fpsyg.2020.01104

**Published:** 2020-05-28

**Authors:** Biruk K. Metaferia, Judit Futo, Raechel Drew, Zsofia K. Takacs

**Affiliations:** ^1^Doctoral School of Psychology, ELTE Eötvös Loránd University, Budapest, Hungary; ^2^Institute of Psychology, ELTE Eötvös Loránd University, Budapest, Hungary; ^3^Centre for Infant Cognition, Department of Psychology, The University of British Columbia, Vancouver, BC, Canada; ^4^Institute of Education, ELTE Eötvös Loránd University, Budapest, Hungary

**Keywords:** executive function, parental belief, home activity, preschool, play

## Abstract

The purpose of the present study was to replicate and extend previous findings that depict a link between preschoolers’ home experience and EFs. It also examined Hungarian parents’ views about the purpose of preschool education and its relationship with their play beliefs. A total of 87 Hungarian preschoolers participated in neuropsychological testing of executive functioning (44 boys, 42 girls, one not reported; mean age = 62.37 months; SD = 8.33 months; age range = 47–80 months) and their parents (8male and 79 females; mean age = 37.73 years; SD = 5.64 years; age range = 22–63 years) filled in questionnaires. The finding from hierarchical regression analyses depicted that the frequency of pretend play the preschoolers engage in and parental play support beliefs were small to medium-sized predictors of children’s inhibitory control, after accounting for age and SES. Children’s frequency of participation in fine motor activities at home was a small but significant predictor of their visual-spatial working memory, after controlling for age and SES. Furthermore, results indicated that parents hold the belief that the development of social-emotional competence and children enjoying themselves instead of academic skills is the primary purpose of preschool education. To sum up, parental play support and preschoolers’ activities at home are important predictors for children’s EF skills.

## Introduction

Play is a naturally occurring activity that offers children important developmental benefits (LaForett and Mendez, 2016). According to early theories of child development, play is a primary source of learning beyond being a means to healthy social–emotional development (see Piaget, 1972). A substantial body of literature underlines the significant contribution of play in cognitive, language, physical, social, and emotional development (Parmar et al., 2004; Tamis-LeMonda et al., 2004; Burdette and Whitaker, 2005; Johnson et al., 2005; Ginsburg, 2007). Literature also highlights the contribution of aspects of play (e.g., problem-solving) to the development of executive functions (EF) in early childhood (e.g., Burdette and Whitaker, 2005). However, there has been fairly little research on the topic.

Executive functions is a term used to describe a set of cognitive skills that enable the conscious control of thought and action in order to reach as specific goal (Jurado and Rosselli, 2007). It consists of three distinct, but interrelated core components: inhibitory control, cognitive flexibility, and working memory or updating (Miyake et al., 2000). Inhibitory control refers to the skill to disregard disruptive stimuli and undesired responses in favor of a target stimuli (Bull and Scerif, 2001; Monette et al., 2015). Cognitive flexibility is the ability to flexibly switch between mental sets in order to adjust one’s thinking and behavior to accommodate changes in the environment (Best et al., 2009). Working memory (WM) refers to the capacity to actively hold relevant information in mind and perform operations on it for a brief period of time (Cragg and Gilmore, 2014; Monette et al., 2015). WM can further be divided into verbal and visual–spatial components (Diamond, 2013).

There is also a growing body of literature indicating links between EF and various dimensions of school readiness, such as math, science, language, social, and emotional competence (Bull et al., 2008; Ponitz et al., 2009; Nayfeld et al., 2013). A meta-analysis by Duncan et al. (2007) has shown that children’s eventual academic performance in elementary school can be predicted by their EF skills in preschool.

### Children’s Early Experience and Development of Executive Functions

The development of EF skills is heavily influenced by environmental factors in addition to biological maturation (Cicchetti, 2002; Blair, 2006; Vernon-feagans et al., 2016). Accordingly, research has underlined the importance of child-caregiver relationships and environmental conditions in the development of EFs during childhood (e.g., Diamond, 2013; Yu and Smith, 2016). A recent meta-analysis has indicated that parental warmth is positively associated with their children’s EF skills (Valcan et al., 2017).

Play provides an opportunity for children to develop and refine their EF skills. It allows children to experiment, solve problems, cooperate with others and try out different behaviors (Bento and Dias, 2017). Furthermore, play can facilitate the development of perspective-taking and help children to develop their emotional and behavioral regulation (Klugman and Smilansky, 1990; Isenberg and Jalongo, 2001). For example, through play children can learn to take turns and gain experience of expressing and regulating strong emotions (Fantuzzo et al., 1998).

### Parental Play Beliefs, Children’s Play Experiences and Developmental Outcomes

Important developmental theories (e.g., Piaget and Vygotsky) claim that play is a vital developmental activity rather than a luxury (Duncan and Lockwood, 2008). However, even today, parents’ opinions range from considering play as an important means of development to simply a form of amusement (Farver and Howes, 1993; Fogle and Mendez, 2006; Fisher et al., 2008). Accordingly, a validation study by Fogle and Mendez (2006) produced two distinct factors concerning parents’ beliefs about play. The first factor (“Play Support”) represents the parental belief that play is an important developmental activity for children, beyond entertainment. Parents with a strong play support belief hold the view that they can teach their children various skills such as social skills or behavior regulation skills via play. The second factor (“Academic Focused”) represents the belief that amusement is the sole purpose of play. Parents with “Academic Focused” beliefs stress the importance of explicit academic activities, such as reading to the child. They tend to believe that play is a less valuable activity with regards to child development.

A growing body of literature emphasizes the importance of parental play beliefs in relation to parents’ engagement in their children’s play and the support they provide during their children’s play (Ihmeideh, 2019) as well as the ways in which parents arrange learning environments and create opportunities for play in the home (Farver and Wimbarti, 1995; Haight et al., 1997). Parents who acknowledge the value of play in development are more likely to facilitate it by actively engaging in and encouraging children’s play, and supporting peer play (Farver and Howes, 1993; Farver et al., 1995; Haight et al., 1997; Parmar et al., 2004). Moreover, parents endorsing the developmental significance of play see their participation and involvement in their children’s play as necessary (Haight et al., 1997). Further, the literature shows that parental engagement in their children’s play is associated with the acquisition of prosocial behaviors (Putallaz, 1987), improvement in cognitive skills (Lin and Yawkey, 2013), and better emotional regulation (O’brien and Md-Yunus, 2007).

Currently there are also investigations demonstrating a positive association among parental play beliefs, children’s play skills and developmental outcomes. For instance, studies found that parents’ play support beliefs were positively associated with their children’s interactive play skills (Fogle and Mendez, 2006; LaForett and Mendez, 2016), while parents’ academic focused beliefs were negatively associated with the same set of skills (LaForett and Mendez, 2016). An investigation by Lin and Yawkey (2014) indicated that parental play beliefs were associated with children’s social skills even after controlling for the parental background variables associated with children’s social competence. Parker et al. (1999) found that children of parents endorsing the developmental importance of play gain more cognitive competencies and independence than children of parents who do not hold these values. In sum, children of parents strongly valuing the importance of play and providing support for it tend to have better developmentally important skills (Lin and Yawkey, 2014).

### Present Study

Several studies have investigated the contribution of household activities (e.g., parenting practice, scaffolding, child play) to child development. Most of these studies have focused on the contribution of these activities to a child’s physical, cognitive, social, and emotional development (Hughes and Ensor, 2009; Bernier et al., 2010, 2012; Rhoades et al., 2011; Blair et al., 2014). Despite the availability of literature highlighting the importance of play in children’s EF development (see Burdette and Whitaker, 2005), very few studies have specifically focused on this topic. In addition, most existing studies focus on developmental outcomes associated with specific forms of play, primarily pretend play (e.g., Carlson et al., 2014; Thibodeau et al., 2016). The developmental implications of other forms of play (such as peer play, solitary play, motor play) and further home activities, including fine motor activities, arts and crafts, screen time, and night sleeping time have received limited attention in the literature. The present investigation aims to fill this gap.

As discussed, parental play beliefs are important in that they may impact the extent to which parents facilitate and support their children’s play at home (Farver and Wimbarti, 1995; Haight et al., 1997; Avornyo and Baker, 2018; Ihmeideh, 2019). Even with increasing interest in parental play beliefs within different socio-cultural contexts (Avornyo and Baker, 2018), only a few investigations have examined the link between parents’ play beliefs and their children’s developmental outcomes. Lin and Yawkey (2014) found that parental play beliefs demonstrated a significant, positive relationship with their children’s social competence, even after controlling for parental background variables typically associated with children’s social competence. Metaferia et al. (2020) found that parental play support belief is a medium sized predictor of Ethiopian preschoolers’ inhibitory control after accounting for preschoolers’ age and family SES. To our knowledge, the present study is only the second study that has investigated the link between parents’ play beliefs and preschoolers’ EF, this time in a Central European country, Hungary.

The purpose of the current study was to replicate and extend previous findings (Metaferia et al., 2020) which indicate a link between preschoolers’ home experiences (parental play beliefs and preschoolers’ home activities) and their EF skills, and was first conducted in Ethiopia. The current study examines Hungarian parents’ beliefs about the purpose of preschool education and its relationship with their play beliefs. The following research questions were investigated in the study:

• Are there associations between parents’ play beliefs and their children’s EF skills?
• Do preschoolers’ frequency of engagement in activities at home (including play and other activities) significantly relate to their EF skills?
• What beliefs do Hungarian parents hold about the purpose of preschool education and how are their educational beliefs related to their play beliefs?

Based on the accumulating literature, we hypothesized that the frequency and duration of play activities and parents’ play support would be related to children’s EF skills. More specifically, based on the findings from our previous study (Metaferia et al., 2020), we hypothesized that parental play support beliefs would be related to children’s inhibitory control skills, with strong positive play support beliefs associated with better inhibitory control skills. We also speculated based on this previous study that children’s frequency of participation in arts and crafts activities would be positively linked to their VSWM skills. On the other hand, we did not make specific hypotheses concerning the parents’ beliefs of the primary purpose of preschool education, this was an exploratory question of the present study.

## Materials and Methods

### Participants

The sample comprised eighty-seven preschoolers (44 boys, 42 girls, and one not reported; mean age = 62.37 months; SD = 8.33 months; age range = 47–80 months) and their parents (eight male and 79 females; mean age = 37.73 years; SD = 5.64 years; age range = 22–63 years) from four preschools in Budapest (the capital of Hungary) and one preschool in Sopronhorpács (a Hungarian village). Eleven of the 87 participants were from Sopronhorpács. Independent-samples *t*-tests depicted that there were no significant differences between the two subsamples on either children’s age (*M*_Budapest_ = 61.90, *SD*_Budapest_ = 8.26; *M*_Sopronhorpács_ = 65.45, *SD*_Sopronhorpács_ = 8.54); *t*(82) = −1.32, *p* = 0.189; or SES (*M*_Budapest_ = 0.028, *SD*_Budapest_ = 0.797; *M*_Sopronhorpács_ = −0.070, *SD*_Sopronhorpács_ = 0.847); *t*(82) = 0.376, *p* = 0.708; and other important variables such as EF scores and play beliefs. The caregivers who filled out the questionnaire were the children’s mothers (93.1%), fathers (5.7%), and grandmothers (1.1%). The child participants had been attending preschool for less than a year (50.6%), between one and two (47.1%), and for 3 years (2.3%) at the time of data collection. The education level of parents is summarized in Table 1.

**TABLE 1:** Sample demographics.

**Education level**	**Mother’s**		**Father’s**	
	**Frequency**	**Percent**	**Frequency**	**Percent**
Elementary school complete	3	3.4	4	4.6
High school complete	23	26.4	36	41.4
College diploma	26	29.9	15	17.2
University degree	30	34.5	25	28.7
Graduate degree (master’s or above)	5	5.7	6	6.9
Not reported	–	–	1	1.1

### Procedure

Following approval of the study by the Research Ethical Committee of the Faculty of Psychology and Education at ELTE Eötvös Loránd University (issue number: 2017/209), the directors of a number of preschools were contacted. After receiving confirmation of interest from the directors, and obtaining consent from parents, questionnaires were sent home with children. Parents were requested to return completed questionnaires to the preschool, where the researchers collected them. In addition, trained research assistants administered EF tests to the participating children during individual testing sessions in a quiet room at their preschool. The order of the neuropsychological tests was the same for all participants: starting with the go/no-go task, followed by the switching task, and finishing with the visual-spatial working memory task. The session took about 20–25 min.

### Measures

The questionnaire consisted of four parts: demographic information, parental beliefs regarding the purpose of preschool education, play beliefs, and the child’s home activities. It was prepared in English and translated into Hungarian. The translation process involved two independent translators who were native speakers of Hungarian and fluent in English. The second and third authors collected the two translations, checked the discrepancies and made the final version accordingly.

#### Demographic Information

Information concerning the respondent’s relationship with the child, the child’s gender and age, the highest educational level of the parents, the family gross annual income, family size, and preschoolers’ year of enrollment in preschool was collected. Parental educational level was measured on a five-point scale ranging from elementary school completed (1) to graduate degree/Master’s or above (5). The family’s annual gross income was measured on a nine-point categorical scale. An aggregate SES variable was created by averaging the standardized score of the average education of the parents and the standardized score of the annual gross income per family member.

#### Parental Play Beliefs Scale

The Parental Play Beliefs Scale (PPBS), developed by Fogle and Mendez (2006), was used to measure parents’ play beliefs. Originally, PPBS was used with African-American mothers of preschoolers in the Head Start program. The scale consists of 25 Likert-scale items with five responses ranging from 1 (disagree) to 5 (very much agree). A principal component analysis in the validation study (Fogle and Mendez, 2006) revealed two subscales: Play Support and Academic Focused beliefs, the first consisting of 17 items (e.g., “through play my child develops new skills and abilities,” and “I can help my child learn to control his or her emotions during play”) and the second including 8 items (e.g., “I do not think my child learns important skills by playing,” and “I would rather read to my child than play together”). The subscales showed a good level of internal consistency (α = 0.90 and 0.73, respectively) in the scale validation study (Fogle and Mendez, 2006), as well as in the present study (α = 0.80 and 0.70, respectively) after removing one item from the play support subscale and two items from the academic focused subscale. The bivariate correlation of the two subscales showed a strong negative correlation (*r* = -0.52, *p* < 0.001).

#### Parents’ Beliefs About the Purpose of Preschool Education Scale

To measure parents’ beliefs about the purpose of preschool education, a rank-order scale consisting of seven items indicating different possible purposes of preschool education was developed by the present investigators. The items were: general knowledge, cognitive skills, language development, social skills, enjoying themselves, emotional well-being and academic skills. Parents were requested to rank the items based on their personal beliefs on the importance starting from 1 (most important) to 7 (least important).

#### Child’s Home Activities Scale

The Child’s Home Activities Scale (CHAS) consisted of two parts: (1) the frequency of the activity (a Likert-scale) and (2) estimation of duration of time spent on the activities. The Likert scale was developed by Metaferia et al. (2020) to assess the frequency of the preschoolers’ participation in selected activities at home. It consisted of ten items designed to measure the frequency [ranging from “very rarely/less than once a week” (1) to “very frequently/most of the time during the day” (5)] with which preschoolers engage in the following activities: breakfast at home, spending meal time with family, academic-related activities, pretend play, motor play, fine motor activities, arts and crafts, solitary play, peer play, and sports and physical activity. The second part of the CHAS was designed to measure the average duration children are engaged in the activities at home. Parents were provided with five activities and requested to provide an estimation of time (in minutes) their children spent participating in each activity. The time estimations were made separately for each activity for the weekdays and for weekends. The activities examined were: screen time, night sleeping, peer play, playing with adults, and playing alone. Average play duration per a day variable was created for each activity by averaging the weekdays and weekends play time. Average total play duration variable was created by taking the average play duration per a day for peer play, play with adult, and solitary play.

#### Executive Functions Measures

Executive function skills including inhibitory control, switching, and visual-spatial working memory (VSWM) were measured using a computerized go/no-go task, switching task (using PsychoPy 1.85.1 version Psychological Software Tools; Peirce, 2008) and the Mr. Peanut task, respectively.

#### Inhibitory Control

A computerized go/no-go task using pictures of a fish and a shark (see Wiebe et al., 2012) consisting of 40 go (fish) and 20 no-go (shark) test trials was used to measure the inhibitory control skills of children. Before starting, children were instructed to press the space bar on a laptop as quickly as possible at the appearance of the target stimulus and withhold pressing the key whenever the non-target stimulus appeared on the screen. Children completed a practice session consisting of 4 go and 2 no-go trials prior to the testing session. During the test, each stimulus appeared on the screen for 1,500 ms unless the child pressed the response key sooner. Based on signal detection theory, sensitivity (*d’*) was calculated for each child using their frequency of responding to go trials/hit/and no-go trials/false alarm/(see Macmillan and Creelman, 2004). The maximum score a child could obtain is 4.20.

#### Cognitive Flexibility

The switching task used in the present study was similar to the task used by Metaferia et al. (2020). A modified Go/No-Go task was used including 4 blocks, in each of which the rule was switched. The task consisted of 16 go and 8 no-go trials in each block. Each stimulus was visible on the screen for 1,200 ms unless the child pressed the response key earlier. The Go and No-Go stimuli were counterbalanced across blocks. Accordingly, a picture of a cat was the go stimuli and a picture of the tiger the no-go stimuli in the first and third blocks; and the stimuli were reversed for the second and fourth blocks. Before the beginning of each block children were informed of the Go and No-Go stimuli by the research assistant. Children completed a practice session before the first block consisting of 4 Go and 2 No-Go trials prior to they start testing. In order to calculate a switching score for each child, sensitivity was first calculated for each child during each block and then a sensitivity difference score between switching blocks (sensitivity of block 3 minus block 1 and sensitivity of block 4 minus block 2) was calculated. Next, the switching score for each child was calculated by taking the average of the difference between switching blocks (i.e., the average of block 3 minus block 1 and block 4 minus block 2). A child could obtain a maximum of 6.08 score.

#### Visual-Spatial Working Memory

The Mr. Peanut test, developed based on the work of Morra (1994), was used in the present study to measure children’s visual-spatial working memory (VSWM). Mr. Peanut is a clown-like figure who appears on the screen decorated with stickers at any of the 14 different body locations after which he disappears. When he reappears the stickers are missing and the children’s task is to indicate the parts of Mr. Peanut that were marked with stickers prior to his disappearance. The test consists of levels ranging from 1 to 7 body parts with three different trials at each level. For the child to move to the next level he/she had to respond to at least one of the three trials correctly. Responding only to one of the three trials correctly resulted in a score of 0.33 and responding at least two of the three trials correctly lead to a score of 1. The total score was the sum of the scores the child received at the different level. The possible maximum score that a child could achieve is 7.00. Children participated in a practice session consisting of three trials prior to the testing session. A child needed to respond to all three practice trials correctly before moving on to the test.

### Data Analyses

A bivariate Pearson’s correlation analyses were used to examine whether there was a significant relationship between parental play beliefs and preschoolers’ home activities on the one hand and preschoolers’ EFs scores on the other. We also used hierarchical regression analyses to determine the extent to which parental play beliefs and preschoolers’ home activities predicted preschoolers’ EF skills. All hierarchical regressions applied in the study used a two-step process. Accordingly, in the first step, sociodemographic variables (children’s age and SES) were entered as controls. In the second step, based on the results of the bivariate correlation analyses, significant correlates of the dependent variable from parents’ play beliefs and preschoolers’ home activity variables were entered together as predictors. Finally, parents’ beliefs regarding the purpose of preschool education were analyzed using a Friedman-test. *Post hoc* analysis with Wilcoxon signed-rank tests were also conducted to examine the significance of the differences in beliefs. The relationship between parental beliefs about the purpose of preschool education and play beliefs was examined using a Kendall’s tau-b correlational analysis.

All statistical analyses were conducted using SPSS v25.0 for Windows (IBM Corporation, Armonk, NY, United States). To manage the normality of the frequency of participation in motor play activity, the scores were managed by reverse score transformation. Accordingly, each score was first subtracted from the highest possible score plus one and then log transformed. Finally, the reversed log transformed scores were reversed back for easier interpretation (see Field, 2009). Furthermore, 8 outliers from mealtime scores and one from play with adult were excluded from the analysis as the transformation didn’t work out.

## Results

Table 2 presents descriptive statistics for the variables used in the analyses.

**TABLE 2:** Mean, standard deviation, minimum and maximum scores on children’s EF performance, home activities and parental play beliefs.

**Variables**	***N***	**Mean**	**SD**	**Minimum– Maximum**
**Child’s EF**				
Inhibitory control	85	2.87	0.86	0.77–4.20
Cognitive flexibility	84	–0.18	0.65	−2.16–1.53
VSWM	86	2.56	0.84	0.67–4.33
**Child’s home activity**				
Academic skills practice after preschool	85	2.69	1.42	1–5
Mealtime together with family	87	3.97	1.07	1–5
Breakfast at home	87	3.76	0.79	2–5
Engage in pretend play	86	3.38	1.43	1–5
Engage in motor play	84	4.15	1.17	1–5
Engage in fine-motor activities	86	3.57	1.15	1–5
Participate in arts and crafts	87	2.43	1.15	1–5
Engage in solitary play	83	3.54	1.21	1–5
Play with peers	86	4.01	1.33	1–5
Do sports and physical activities	86	3.07	1.29	1–5
Screen time	70	1.08h	0.72h	0.05–3.07 h
Night sleep total time	81	9.54h	0.89h	7.65–12 h
Time spent on playing with adult	80	1.83h	1.23h	0.28–5.15 h
Total play time	52	4.96h	2.21h	1.1–9.87 h
**Parents’ play beliefs**				
Parental belief: Academic focused	80	10.36	2.77	6–19
Parental belief: Play support	82	69.78	5.11	55–79

### Correlational Analyses

The first two research questions were designed to examine the association between children’s activity at home and parental play beliefs on one hand and their EFs on the other. Table 4 indicates the results of these association. We also aimed to investigate how parental beliefs about the purpose of preschool education are related to their play beliefs. Surprisingly, as can be seen from Table 5 neither parents’ academic focused beliefs, nor their play support beliefs were significantly correlated with any of the preschool purpose variables.

**TABLE 3:** The median and interquartile range of parental beliefs about the purpose of preschool education.

**Purpose of preschool education**	**Median**	**Interquartile range**
General knowledge	5.00	4.00–6.00
Cognitive skills	4.00	3.75–6.00
Language development	5.00	4.00–6.00
Social skills	2.00	1.00–3.00
Enjoy themselves	2.00	2.00–4.00
Emotional well-being	2.50	1.00–4.00
Academic skills	6.00	4.00–7.00

**TABLE 4:** Bivariate correlations among demographics, home activities, parental play beliefs, and executive functions.

	**1**	**2**	**3**	**4**	**5**	**6**	**7**	**8**	**9**	**10**	**11**	**12**	**13**	**14**	**15**	**16**	**17**	**18**	**19**	**20**	**21**
1. Child’s Age	1																				
2. SES	0.03 *N* = 81	1																			
3. Visual-spatial working memory	0.33** *N* = 83	0.27* *N* = 83	1																		
4. Inhibitory control	0.31** *N* = 82	0.32** *N* = 82	0.40** *N* = 84	1																	
5. Cognitive flexibility	0.20 *N* = 81	0.26* *N* = 81	0.32** *N* = 83	0.32** *N* = 83	1																
6. Academic- related activities	0.25* *N* = 82	−0.04 *N* = 82	0.08 *N* = 85	0.09 *N* = 83	−0.09 *N* = 82	1															
7. Mealtime with family	0.15 *N* = 76	−0.02 *N* = 76	0.11 *N* = 79	0.15 *N* = 78	0.10 *N* = 76	−0.11 *N* = 78	1														
8. Breakfast at home	0.19 *N* = 84	0.35** *N* = 84	0.21 *N* = 86	0.27* *N* = 85	0.15 *N* = 84	−0.09 *N* = 85	0.26* *N* = 78	1													
9. Pretend play	0.06 *N* = 83	0.22* *N* = 84	0.26* *N* = 85	0.35** *N* = 84	0.24* *N* = 83	0.06 *N* = 84	0.18 *N* = 78	0.15 *N* = 86	1												
10. Motor play	0.22 *N* = 81	0.09 *N* = 81	0.16 *N* = 83	0.17 *N* = 82	0.17 *N* = 81	−0.05 *N* = 82	0.20 *N* = 76	0.18 *N* = 84	0.14 *N* = 83	1											
11. Fine motor activities	0.10 *N* = 83	0.18 *N* = 84	0.35** *N* = 85	0.30** *N* = 84	0.09 *N* = 83	0.06 *N* = 84	0.17 *N* = 78	0.25* *N* = 86	0.37** *N* = 85	0.07 *N* = 83	1										
12. Arts and crafts	0.10 *N* = 84	0.17 *N* = 84	0.23* *N* = 86	0.08 *N* = 85	0.08 *N* = 84	0.11 *N* = 85	0.03 *N* = 87	0.09 *N* = 87	0.16 *N* = 86	0.08 *N* = 84	0.54** *N* = 86	1									
13. Solitary play	0.04 *N* = 80	−0.04 *N* = 81	0.16 *N* = 82	0.11 *N* = 81	−0.09 *N* = 80	0.03 *N* = 81	0.24* *N* = 75	0.23* *N* = 83	0.35** *N* = 82	0.25* *N* = 80	0.25* *N* = 83	0.06 *N* = 83	1								
14. Peer play	0.06 *N* = 83	0.18 *N* = 83	0.02 *N* = 85	0.33** *N* = 84	0.13 *N* = 83	0.05 *N* = 84	0.27* *N* = 79	0.07 *N* = 86	0.28** *N* = 85	0.16 *N* = 83	0.23* *N* = 85	0.11 *N* = 86	−0.02 *N* = 82	1							
15. Sports and physical activities	0.10 *N* = 83	0.17 *N* = 83	0.12 *N* = 85	0.22* *N* = 84	0.15 *N* = 83	−0.01 *N* = 84	0.17 *N* = 78	0.33** *N* = 86	0.36** *N* = 85	0.27* *N* = 83	0.21 *N* = 85	0.15 *N* = 86	0.18 *N* = 82	0.40** *N* = 85	1						
16. Screen time	−0.02 *N* = 67	−0.34** *N* = 68	0.00 *N* = 69	−0.04 *N* = 68	0.08 *N* = 67	0.05 *N* = 68	0.14 *N* = 64	0.02 *N* = 70	−0.22 *N* = 69	0.03 *N* = 67	0.12 *N* = 70	0.02 *N* = 70	−0.07 *N* = 67	−0.08 *N* = 70	−0.06 *N* = 69	1					
17. Sleep during night	0.18 *N* = 78	0.20 *N* = 78	0.21 *N* = 80	0.20 *N* = 79	0.19 *N* = 78	−0.23* *N* = 79	0.14 *N* = 73	0.20 *N* = 81	0.11 *N* = 80	0.06 *N* = 78	0.24* *N* = 80	0.14 *N* = 81	0.18 *N* = 77	0.09 *N* = 80	0.06 *N* = 80	0.02 *N* = 70	1				
18. Play with adult	0.06 *N* = 77	0.12 *N* = 79	0.08 *N* = 80	0.16 *N* = 78	0.22 *N* = 77	0.06 *N* = 79	0.26* *N* = 74	0.15 *N* = 80	0.13 *N* = 79	0.15 *N* = 77	0.26* *N* = 80	0.32** *N* = 80	−0.02 *N* = 77	0.09 *N* = 79	0.27* *N* = 79	0.24 *N* = 67	0.27* *N* = 75	1			
19. Total play time	0.18 *N* = 51	0.15 *N* = 50	0.04 *N* = 52	0.33* *N* = 50	0.20 *N* = 50	−0.04 *N* = 52	0.21 *N* = 52	0.23 *N* = 52	0.13 *N* = 51	0.09 *N* = 49	0.26 *N* = 52	0.32* *N* = 52	−0.08 *N* = 49	0.28* *N* = 52	0.29* *N* = 52	0.41** *N* = 50	0.22 *N* = 51	0.80** N = 51	1		
20. Parental belief: Academic focused	−0.02 *N* = 77	−0.32** *N* = 78	−0.05 *N* = 80	−0.10 *N* = 78	−0.05 *N* = 77	0.30** *N* = 80	−0.03 *N* = 73	−0.17 *N* = 80	−0.19 *N* = 79	0.01 *N* = 77	−0.12 *N* = 79	−0.01 *N* = 80	0.03 *N* = 76	−0.18 *N* = 79	−0.09 *N* = 79	0.24 *N* = 65	−0.30* *N* = 74	−0.18 N = 76	−0.11 N = 50	1	
21. Parental belief: Play support	0.13 *N* = 79	0.26* *N* = 79	0.08 *N* = 81	0.40** *N* = 80	0.22 *N* = 79	−0.19 *N* = 80	0.15 *N* = 74	0.11 *N* = 82	0.20 *N* = 81	0.02 *N* = 79	0.11 *N* = 81	0.10 *N* = 82	0.06 *N* = 79	0.14 *N* = 81	0.07 *N* = 82	−0.09 *N* = 66	0.03 *N* = 77	0.30* N = 75	0.38** N = 50	−0.52** N = 76	1

*p < 0.05; **p < 0.001.

**TABLE 5:** Kendall’s tau-b correlations among parental play beliefs and views about the purpose of preschool education variables.

	**1**	**2**	**3**	**4**	**5**	**6**	**7**	**8**	**9**
(1) Academic skills development	1								
(2) Emotional well-being	−0.22* *N* = 82	1							
(3) Enjoy themselves	−0.11 *N* = 82	0.15 *N* = 82	1						
(4) Social skills development	0.18* *N* = 82	−0.43** *N* = 82	−0.43** *N* = 82	1					
(5) Language development	−0.11 *N* = 82	−0.12 *N* = 82	−0.17 *N* = 82	0.11 *N* = 82	1				
(6) Cognitive skills development	−0.20* *N* = 82	0.02 *N* = 82	−0.11 *N* = 82	−0.11 *N* = 82	0.03 *N* = 82	1			
(7) General knowledge	−0.04 *N* = 82	−0.17 *N* = 82	−0.07 *N* = 82	−0.03 *N* = 82	−0.14 *N* = 82	0.04 *N* = 82	1		
(8) Parental belief: Academic focused	0.01 *N* = 75	0.09 *N* = 75	0.03 *N* = 75	0.02 *N* = 75	−0.02 *N* = 75	0.04 *N* = 75	−0.11 *N* = 75	1	
(9) Parental belief: Play support	0.09 *N* = 78	−0.02 *N* = 78	−0.04 *N* = 78	−0.02 *N* = 78	0.08 *N* = 78	0.10 *N* = 78	0.04 *N* = 78	–	1

*p < 0.05; **p < 0.001.

### Hierarchical Regression Analyses

Three hierarchical regression analyses were run to investigate the extent to which preschoolers’ home activity and their parents’ play beliefs were predictive of preschoolers’ EF skills (inhibitory control, visual-spatial working memory, and switching). In each regression analysis, in the first step sociodemographic variables (i.e., child’s age, SES) that had a significant relationship with the corresponding components of EF were controlled, while in the second step the significant correlates of the dependent variables from the parental beliefs and the home activity variables were entered as predictors. The results of the hierarchical regression analyses are summarized in Table 6.

**TABLE 6:** Hierarchical multiple regression models summary predicting inhibitory control, visual-spatial working memory and switching.

	**Inhibitory control**				**Visual-spatial WM**				**Switching**			
	**Step 1**		**Step 2**		**Step 1**		**Step 2**		**Step 1**		**Step 2**	
	β	*95% CI*	β	*95% CI*	β	*95% CI*	β	*95% CI*	β	*95% CI*	β	*95% CI*
Age	0.29	(0.008,0.052)*	0.24	(0.004,0.045)*	0.31	(0.011,0.053)**	0.29	(0.009,0.049)**				
SES	0.30	(0.095,0.574)**	0.15	(−0.061,0.402)	0.27	(0.064,0.502)*	0.21	(0.007,0.439)*	0.26	(0.038,0.403)*	0.21	(−0.009,0.366)
Pretend play			0.25	(0.026,0.278)*							0.19	(−0.014,0.187)
Play support			0.30	(0.016,0.086)**								
Fine motor							0.27	(0.050,0.353)*				
*F*	8.04**		9.12***		8.10**		8.16***		5.76*		4.41*	
*R*^2^	0.19		0.35		0.17		0.24		0.07		0.10	
Adjusted *R*^2^	0.16		0.31		0.15		0.21		0.06		0.08	
*R*^2^ change	0.19		0.16		0.17		0.07		0.07		0.03	

*p < 0.05; **p < 0.01; ***p < 0.001; 95% CI.

In the first regression model preschoolers’ inhibitory control skills were predicted. Age and SES were entered in the first block. As shown in Table 6, age and SES together accounted for a significant level of the variance (19%) [*F*(2,73) = 8.039, *p* = 0.001] in inhibitory control. Then, based on the result of the bivariate correlational analyses, frequency of breakfast at home, pretend play, fine motor activities, peer play, and sports and physical activities in addition to total play time and parental play support beliefs were entered into the regression model in the second block (see Supplementary Appendix for the model containing all predictors). Only the frequency of pretend play and parental play support belief were significant predictors in the model). After removing the non-significant predictors (breakfast at home, fine motor activities, peer play, sports and physical activities, and total play time), the model explained a significant amount of variance of 35% in inhibitory control [*F*(4,73) = 9.123, *p* < 0.001]. As shown in Table 6, parental play support and preschoolers’ frequency of participation in pretend play were found to be medium (β = 0.30) and small-sized (β = 0.27) positive predictors, respectively.

The second hierarchical regression analysis examined the contribution of home activities and parental play beliefs to VSWM. Sociodemographic variables (age and SES) were entered in the model in the initial block, explaining 17% of the variance in VSWM [*F*(2,79) = 8.10, *p* = 0.001]. Next, based on the results of the correlational analyses, frequency of pretend play, fine motor activities and arts and crafts were entered in the model in the second block. However, only frequency of participation in fine motor activities was a significant predictor of VSWM, while pretend play and arts and crafts were not. After removing the non-significant predictors, the total variance explained by the model reached 24% [*F*(3,79) = 8.16, *p* < 001)]. The inclusion of the fine motor activity variable improved the model by 7% and this variable was a significant, small-sized (β = 0.27) positive predictor in the final model, as shown in Table 6.

The third hierarchical regression analysis was built to predict children’s switching skills. In step 1, SES was included as a control variable and explained a significant level of variance (7%) in switching score [*F*(1,80) = 5.76, *p* = *0.019*)]. In step 2, frequency of participation in pretend play was entered into the model. However, the inclusion of this variable did not result in any significant improvement to the model and it was not a significant predictor either.

### Friedman ANOVA

In the third research question, we targeted to investigate the beliefs parents hold about the purpose of preschool education. Friedman ANOVA and Wilcoxon signed-rank tests were used to examine differences between parents’ beliefs about the purpose of preschool education. Overall, there was a statistically significant difference among parents’ beliefs about the purpose of preschool education χ2(6) = 157.57, *p* < 0.001. *Post hoc* analysis with Wilcoxon signed-rank tests using Bonferroni correction, resulting in a significance level set at *p* < 0.0024 was carried out. The median and interquartile range of parental beliefs about the purpose of preschool education is summarized in Table 3. There were statistically significant differences between the following parental beliefs: general knowledge was considered less important as compared to social skills (*Z* = −6.48, *p* < 0.001), enjoying themselves (*Z* = −5.57, *p* < 0.001), and emotional well-being (*Z* = −5.21, *p* < 0.001); cognitive skills was considered less important as compared to social skills (*Z* = −6.35, *p* < 0.001), enjoying themselves (*Z* = −4.54, *p* < 0.001), and emotional well-being (*Z* = −4.82, *p* < 0.001); language development was considered less important as compared to social skills (*Z* = −6.80, *p* < 0.001), enjoying themselves (*Z* = −4.92, *p* < 0.001), and emotional well-being (*Z* = −5.06, *p* < 0.001); academic skills was considered less important as compared to social skills (*Z* = −7.25, *p* < 0.001), enjoying themselves (*Z* = −5.78, *p* < 0.001), and emotional well-being (*Z* = −5.65, *p* < 0.001). However, there were no statistically significant differences between the rest [general knowledge vs. cognitive skills (*Z* = −2.25, *p* = 0.025), general knowledge vs. language (*Z* = −1.15, *p* = 0.252), general knowledge vs. academic skills (*Z* = −1.36, *p* = 0.173), cognitive skills vs. language development (*Z* = −1.06, *p* = 0.288), cognitive skills vs. academic skills (*Z* = −3.01, *p* = 0.003), language development vs. academic skills (*Z* = −2.13, *p* = 0.033), social skills vs. enjoying themselves (*Z* = −2.04, *p* = 0.042), social skills vs. emotional well-being (*Z* = −2.28, *p* = 0.023), and enjoying themselves vs. emotional well-being (*Z* = −0.13, *p* = 0.901)].

## Discussion

The main goal of the present study was to examine the role of play and other home activities in children’s developing EF skills in a Hungarian sample. This was achieved with parental reports of the frequency and duration with which their children engage in different sorts of play at home and also by investigating parental play beliefs. Additionally, we aimed to assess parents’ beliefs about the purpose of preschool education and examine how these educational beliefs are related to their beliefs about play.

Small-to-moderate positive correlations were found between the three components of EF skills, which is in line with the literature (see Lan et al., 2011) suggesting that these components are related but separable components. Our results demonstrated that parental play support and children’s frequency of pretend play are significant predictors of inhibitory control, after controlling for age and SES. This means that children who frequently engage in pretend play at home and have parents who hold strong play support beliefs are likely to have better inhibitory control skills than their peers. The result that parental play support is a medium-sized predictor of children’s inhibitory control skills after accounting for age and SES is consistent with the finding reported by Metaferia et al. (2020) in an Ethiopian sample. Additionally, frequency of pretend play was also an important predictor for inhibitory control. This replicated previous finding (Kelly and Hammond, 2011) indicating that pretend play is positively linked to the development of inhibitory control in young children.

Interestingly, unlike in the present study, Metaferia et al. (2020) found that the frequency of having breakfast at home was also a significant predictor of children’s inhibitory control skill. This difference could be attributed to a variation in the socio-cultural context under which the two studies were conducted. Breakfast service is common in Hungarian preschools such that preschoolers are given an opportunity to eat at their preschool center when they arrive in the morning. However, such service is not provided by Ethiopian preschools.

Another important finding in the present study is that the frequency with which children engage in fine motor activities at home was a small but significant predictor of their performance in a VSWM task, after controlling for age and SES. According to our finding, children with better participation in fine motor activities at home demonstrate better visual-spatial working memory performance which is not in support of our hypothesis speculated that frequency of participation is arts and crafts activities (instead of fine motor skills) would have been a significant predictor of VSWM. The results of the current study extend a previous finding indicating that fine motor skills are significantly positively associated with VSWM among typically developing adolescents (Rigoli et al., 2012). In the same line, another study indicates that motor coordination is linked to visuospatial working memory among children with developmental coordination disorder (Alloway and Temple, 2007). On the other hand, even though some home activities were significantly correlated with children’s cognitive flexibility in the present study, none of these variables were significant predictors after controlling for SES.

The result in the present study indicates that Hungarian parents hold the belief that development of social-emotional competence is the primary purpose of preschool education. The reason for this result could be that the purpose of preschool education in Hungary (Kormányrendelet az óvodai nevelés országos alapprogramjáról, Act of 363/2012) is very well articulated and the expectation for a preschool to be academically focused is neither culturally widespread nor accepted (Bevezet and Alapprogram, 2019). However, no significant association was found between the parents’ beliefs about the purpose of preschool education and their play beliefs. This means that parents holding academic focused or play support beliefs do not have a specific corresponding belief about the purpose of preschool education.

The current investigation makes valuable contributions to our understanding of the role of play and other home activities in children’s developing EF skills. However, it has several limitations. First, our findings should be interpreted with caution given that parental play beliefs and preschoolers’ activities at home could vary as a function of different socio-economic contexts. A follow-up study using a cross-cultural design is highly recommended. Second, reliance on self-report data in assessing children’s activities at home and parental play beliefs might make the data susceptible to social desirability bias. Moreover, the frequency with which children engage in different home activities was assessed with single items, thus we have no information regarding the reliability of these measures. Third, the content of home activities was not explored in the present study and may be of greater importance than the frequency of participation in them. Future studies should take the content of home activities into account to gain a more comprehensive understanding of what activities children engage in. Further, IQ was not assessed and may account for some of the variability in the EF scores. Additionally, we used a convenience sample which was not representative of the population from which the participants were recruited. To establish further generalizability of our results, it will be important to recruit a representative sample in follow-up studies. Finally, probably the biggest limitation of the present study is that we applied a correlational strategy thus the associations found might not reflect casual relations and might be affected by confound variables. While we used socioeconomic status as a control variable in all the models, it is still questionable whether other variables might explain the results. It is possible, for instance, that parental play support, children’s play activities and inhibitory control skills are related to other parenting factors or home activities and that such a third variable (e.g., parent-child relationship, attachment security, scaffolding) could explain the relationship (see Bernier et al., 2010, 2012; Hammond et al., 2012; Fay-Stammbach et al., 2014). In a similar vein, the link between frequency of children’s engagement in fine motor activity and visual working memory could be explained by a third variable such as level of motor coordination (see Rigoli et al., 2012).

In sum, the present study has important conclusions regarding the role of play and other activities at home in the development of EF skills. We found that the frequency of pretend play and parental play support beliefs are important predictors of preschoolers’ inhibitory control skills, while the frequency of fine motor activities is a significant factor in visual-spatial working memory. Additionally, we found that Hungarian parents hold the belief that building socio-emotional competence is the primary purpose of preschool education as opposed to developing academic skills. However, these educational beliefs were not related to parents’ views concerning the value of play. Further studies are needed to replicate the present findings.

## Data Availability Statement

The original contributions presented in the study are publicly available. This data can be found here: https://data.4tu.nl/repository/uuid:3a4e6261-abb3-483e-a6a4-407c99ca6db2.

## Ethics Statement

The studies involving human participants were reviewed and approved by the Research Ethical Committee of the Faculty of Psychology and Education at ELTE Eötvös Loránd University (issue number: 2017/209). Written informed consent to participate in this study was provided by the participants’ legal guardian/next of kin.

## Author Contributions

BM, JF, and ZT made important contributions to conception and design, scale development, and analysis and interpretation of data. BM prepared the manuscript. RD, JF, and ZT read the manuscript and provided critical feedback.

## Conflict of Interest

The authors declare that the research was conducted in the absence of any commercial or financial relationships that could be construed as a potential conflict of interest.
