# Supplementary material for: Parents’ Beliefs About Play and the Purpose of Preschool Education, Preschoolers’ Home Activity and Executive Functions
Source: Front Psychol. 2020 May 28;11:1104. doi: 10.3389/fpsyg.2020.01104 (PMC7326145; doi:10.3389/fpsyg.2020.01104)
Supplement: Supplementary file 1 [file Table_1.pdf]

Supplementary Material

Hierarchical multiple regression models predicting inhibitory control, visual-spatial working memory and switching using all predictors

	Inhibitory control				Visual-spatial WM				Switching			
	Step 1		Step 2		Step 1		Step 2		Step 1		Step 2	
	β	*95% CI*	*β*	*95% CI*	*β*	*95% CI*	*β*	*95% CI*	*β*	*95% CI*	*β*	*95% CI*
Age	.31	(.001, .061)*	.25	(-.004, .054)	.31	(.011, .053)**	.29	(.009, .050)**				
SES	.16	(-.157, .517)	-.08	(-.457, .265)	.27	(.064, .502)*	.21	(-.005, .436)*	.26	(.038, .403)*	.21	(-.009, .366)
Breakfast at home			.14	(-.175, .457)								
Pretend play			.31	(-.026, .405)			.08	(-.089, .181)			.19	(-.014, .187)
Peer play			.30	(-.010, .375)								
Fine motor			.05	(-.210, .289)			.25	(-.012, .377)				
Arts and crafts							-.008	(-.182, .170)				
Sports and physical activities			-.07	(-.290, .200)								
Total play time			-.06	(-.003, .002)								
Play support			.24	(-.014, .089)								
F		2.93		2.57*		8.10**		4.89**		5.76*		4.41*
R2		.13		.41		.17		.25		.07		.10
adj R2		.08		.25		.15		.20		.06		.08
R2-change		.13		.28		.17		.08		.07		.03

**p <.05; **p <.01; ***p <.001; 95% CI*
